# Tertiary lymphoid structures in esophageal cancer: a novel target for immunotherapy

**DOI:** 10.3389/fimmu.2025.1543322

**Published:** 2025-06-04

**Authors:** Ke Zhai, Yuting Ma, Xue Gao, Kun Ru, Miaoqing Zhao

**Affiliations:** ^1^ Shandong Cancer Hospital and Institute, Shandong First Medical University and Shandong Academy of Medical Sciences, Jinan, China; ^2^ Department of Pathology, Shandong Cancer Hospital and Institute, Shandong First Medical University and Shandong Academy of Medical Sciences, Jinan, China

**Keywords:** tertiary lymphoid structures, esophageal cancer, tumor microenvironment, immunotherapy, predictive biomarker

## Abstract

Esophageal cancer (EC), especially esophageal squamous cell carcinoma (ESCC), which is the most common subtype in China, is one of the most aggressive gastrointestinal malignancies. Traditional treatments like surgery, radiotherapy, and chemotherapy have limited success, but the study of tertiary lymphoid structure (TLS) has opened new avenues for immunotherapy. TLS is an ectopic immune cell cluster, including B cells, T cells, and dendritic cells (DCs), that forms in chronic inflammation such as tumors. TLS is often found at tumor invasion margins and is linked to better prognosis in EC patients, with higher TLS density and maturation associated with prolonged survival. TLS can also serve as a biomarker to assess immunotherapy outcomes. Further exploration of TLS molecular mechanisms could innovate EC treatments, especially by inducing TLS formation to improve therapy. This review summarizes the characteristics of TLS in EC, clinical findings, and the limitations and directions of current research, offering insights for future immunotherapy strategies.

## 1 Introduction

EC ranks among the most aggressive forms of malignancies within the gastrointestinal system (1). In China, EC ranks fifth both in terms of cancer-related mortality and the incidence rate of male malignancies (2). EC in this measurement specifically refers to ESCC. Recent data from American Cancer Society indicate that the 5-year survival rate stands at 22% of EC (3). At present, the primary clinical interventions for EC consist of surgical resection, radiotherapy, and chemotherapy. Nevertheless, these approaches often yield suboptimal outcomes, largely due to their limited therapeutic efficacy and the significant adverse effects associated with traditional treatments (4). The microenvironment of EC is filled with complex and variable fibrous tissues and a substantial population of cells with immunosuppressive functions. These features provide conditions for tumor cells to spread and evade immune surveillance (5). Immunotherapy is emerging as a promising frontier in the treatment of EC. Currently, clinical trials and research on immunotherapy for EC are being actively conducted (6). A recent study has shown that the combination of carrelizumab and chemotherapy increased the pathological complete response (pCR) rate by 23.3% (28.0% vs. 4.7%) and the R0 resection rate by 85.7% (97.1% vs. 85.7%). This combination significantly improved the prognosis of EC patients while maintaining manageable safety (7). Furthermore, the combination of benmelstobart, anlotinib, and chemotherapy in a four-drug regimen has shown promising efficacy in treating advanced or metastatic ESCC. This regimen achieved a median progression-free survival (mPFS) of 14.9 months, an objective response rate of 72%, and a disease control rate of 84%. These results significantly improve the prognosis of advanced EC and provide a new option for first-line treatment (8). Moreover, toripalimab combined with neoadjuvant chemotherapy has increased the one-year event-free survival rate by 13.6% (77.9% vs. 64.3%). It also improved the one-year overall survival (OS) rate by 11.1% (94.1% vs. 83.0%) and the pCR rate by 14% (18.6% vs. 4.6%) (9). These results clearly demonstrate the significant role of immunotherapy in improving efficacy and the prognosis of EC. Nevertheless, owing to the uniqueness of the microenvironment of EC, therapeutic regimens do not apply to all patients. Drug resistance remains prevalent (10). Additionally, the insufficient function and number of anti-tumor T cells pose significant challenges in extensive immunotherapy (11). Given the immunosuppressive nature of the tumor microenvironment (TME) in EC, exploring structures like TLS that can enhance immune responses becomes particularly crucial.

In 1889, Stephen Paget first proposed the hypothesis that tumor cells grow like “seeds” in a specific microenvironmental “soil” (12). Since then, researchers have expanded the understanding of the TME. It is characterized as a specialized biological niche that supports tumor cells’ survival and promotes their interactions. Further studies revealed that the TME is pivotal in shaping tumor proliferation, drug resistance, invasion, and immune escape (13–15). Studies have emphasized that within the TME, a diverse array of immune cell populations is essential for modulating immune responses against tumors. These populations include T cells, B cells, natural killer (NK) cells, and macrophages (16). NK cells and macrophages are elements of the innate immune response. They exert direct cytotoxic effects on tumor cells. They also facilitate lymphocyte activation within the adaptive immune response by presenting antigens, such as T cells and B cells. This, in turn, enhances anti-tumor activity (17, 18). Tumor-infiltrating lymphocytes (TILs) are targeted autoimmune cells that are present in the TME. They encompass diverse phenotypes of T cells, including the cluster of differentiation (CD) 4+ and CD8+ subsets, along with regulatory T cells (Tregs) and B cells (19). The abundance, type, and functional status of these cells are key in determining the sensitivity of the tumor to the immune response. Within the TME of EC, TLS is regarded as a unique immune tissue structure. It is considered a potential breakthrough for ameliorating the immunosuppressive microenvironment because of its capacity to recruit and activate anti-tumor immune cells. In addition, tertiary lymphoid structures (TLSs) are aberrant lymphoid formations that arise within non-lymphoid tissues in response to persistent inflammation, such as in tumors. These structures consist of a diverse array of immune cells, prominently featuring B and T lymphocytes among others. TLSs, also referred to as tertiary lymphoid organs or ectopic lymphoid structures, represent a distinct class of lymphoid aggregates (20). The frequency of TLS is high in autoimmune diseases and some chronic diseases. It is especially prominent within the synovial tissue of individuals afflicted by rheumatoid arthritis and in suppurative sweats (21). At the same time, the occurrence of TLS has been linked to improved clinical outcomes across multiple tumor types. These include lung carcinoma, cholangiocarcinoma, head and neck squamous cell carcinoma, colorectal carcinoma, breast carcinoma, and pancreatic carcinoma. The presence of TLS usually indicates a better prognosis (22–27). However, in certain immune-related diseases, the immunopotentiating effect of TLS may be associated with a poor prognosis (28).

The presence of TLS in EC tissues is one of the key focuses in the field of tumor immunology research. It affects patient prognosis and may be a novel target for immunotherapy of EC. Therefore, this article reviews the characteristics of TLS composition and relevant clinical results in EC. It also presents the limitations of current studies and future research directions. The aim is to inform and guide future research endeavors focused on TLS in EC.

TILs in EC are primarily made up of T cells, B cells and NK cells, while TLSs in EC are characterized by a predominance of T and B cells (29). This section, therefore, centers on the detailed characterization of T cell and B cell populations within TILs in the esophageal TME.

## 2 EC and TILs

### 2.1 T cells

Extensive research has demonstrated the critical role of T cells in EC immunity. In EC, T cells mainly consist of CD8+ cytotoxic T cells and CD4+ helper T (Th) cells (30). CD4+ T cells further differentiate into subsets such as Tregs and follicular helper T cells (Tfh) (30). Upon encountering antigens, these cells mature into effector populations and migrate to tumor sites to exert their functions (30). CD8+ T cells play a crucial role in antitumor immunity, primarily via the sprouty RTK signaling antagonist 1 pathway. Overexpression of this signaling antagonist in CD8+ T cells can lead to a state known as progenitor-like exhaustion. In this state, the cells lose effectiveness against tumors (31). Besides this pathway, CD8+ T cells utilize cytokines and chemokines such as interferon-γ (IFN-γ), tumor necrosis factor, interleukin (IL) 15, C-X-C motif chemokine ligand (CXCL) 9 and CXCL10 to eliminate tumor cells (31). High numbers of CD8+ T cells within tumors are associated with improved disease-free survival in EC patients (32). Additionally, IFN-γ produced by CD8+ T cells strengthens the antitumor response. It enhances the expression of major histocompatibility complex class I molecules. It also stimulates pro-inflammatory cytokine production and promotes endothelial cell defenses against tumor invasion (32). CD4+ T cells in EC differentiate into subsets including Tregs and Tfh, each performing distinct roles (30). CD4+ T cells can support CD8+ T cell activity through a paracrine mechanism. Specifically, Tfh cells activate DCs by secreting paracrine factors such as Semaphorin-4D. This enhances the antigen presentation function of DCs and promotes the anti-tumor activity of CD8+ T cells (33). Among CD4+ subsets, Tregs generally promote immune suppression and tumor progression. The immunosuppressive microenvironment in EC frequently involves interactions among Tregs, exhausted CD8+ T cells, M2 macrophage (30, 34). Specifically, Tregs secrete IL-10 to act on B cells, possibly through IL-10 receptors, driving their differentiation into IgG4+ plasma cells. IgG4 inhibits CD8+ T cell function through low-affinity interactions between Fc and Fcγ receptors. Together with IL-10, IgG4 also induces M2 macrophage polarization, characterized by CD163 upregulation and IL-1β suppression (30). Furthermore, in EC, Tregs suppress the function of TLS through the lymphocyte-associated protein 4 (CTLA4) pathway. They engage CD80 and CD86 receptors on antigen-presenting cells and inhibit effective immune activation (34). These processes lead to the formation of immunosuppressive TLS and promote tumor progression. Another important subset, Tfh cells, typically exert antitumor effects by facilitating B cell activation and forming TLS (35, 36). Tfh cells promote the formation and maturation of TLSs by secreting CXCL13 and IL-21 (35–37). CXCL13 binds to CXCR5 on B cells and drives their migration into TLSs (35, 36). These processes activate B cells and strengthen antitumor immunity (35, 36). Blocking CXCL13 may impair the function of Tfh cells to recruit B cells and promote TLS formation (38). CXCL13 organizes the core structure of TLSs (35). IL-21 also supports the differentiation of B cells into plasma cells and boosts CTL function (37). Single-cell RNA sequencing revealed that TLS-positive tumors showed higher levels of Tfh cells, B cells, plasma cells, Th17, and Th1 cells (36). These interactions collectively enhanced antitumor responses, particularly in the neoadjuvant immunotherapy group (36). Similar supportive roles of Tfh cells have been demonstrated in other cancers, such as colorectal cancer, nasopharyngeal carcinoma, and ovarian cancer. In these cancers, Tfh cells similarly drive TLS formation and boost antitumor immunity (39–41).

### 2.2 B cells

In TLSs of ESCC, B cells are associated with improved patient prognosis. TLSs provide a specialized microenvironment that supports the maturation, activation, and differentiation of B cells. These processes lead to the formation of antibody-secreting plasma cells, thereby enhancing humoral anti-tumor immunity (42). B cells also function as potent antigen-presenting cells. They facilitate T cell activation and infiltration, particularly in cooperation with CD8+ T cells localized within TLS (32). Clinical studies have demonstrated that EC patients who respond to immune checkpoint blockade (ICB) therapy exhibit significantly higher intratumoral densities of CD20+ B cells compared to non-responders (43). These findings highlight the critical role of B cells in orchestrating effective anti-tumor immune responses and therapeutic outcomes (44, 45). B cell recruitment to TLSs is primarily mediated by the chemokine CXCL13. This chemokine is secreted by CD4+ T cells and guides B cells toward the tumor site, facilitating the assembly of TLSs (35, 36). Once localized within TLSs, B cells receive activation signals through key co-stimulatory receptors. These receptors include tumor necrosis factor receptor superfamily member 9, tumor necrosis factor receptor superfamily member 14 (LIGHT), and CXCR5. These signals promote their differentiation into plasma cells capable of producing tumor-specific antibodies (41, 44). These plasma cells play a vital role in the humoral immune response against tumors. Additionally, follicular B cells within TLSs form germinal centers (GCs). Within these centers, they undergo somatic hypermutation and affinity maturation, enhancing the quality and specificity of the antibody response (36). The contribution of B cells within TLSs extends beyond antibody production. Through antigen presentation and cytokine secretion, activated B cells participate in the local orchestration of immune responses (44). Particularly in the context of ICB therapy, B-cell-rich human leukocyte antigen (HLA) -A+ TLSs have been associated with enhanced therapeutic response in ESCC (44). These findings underscore the functional significance of TLS-resident B cells as both immune effectors and biomarkers of treatment efficacy.

Outside TLSs, B cells in the TME display a context-dependent dual role in modulating tumor progression. On the one hand, tumor-infiltrating B cells with IgG expression are more abundant in cancerous tissues than in adjacent normal tissues. They are linked to favorable clinical outcome (46, 47). Chemotherapy can further enhance their antitumor potential by promoting CD40 signaling and antibody production (48). Increased abundance of plasma cells following PD-1 blockade therapy has been correlated with prolonged survival in ESCC patients. This finding highlights the importance of B cell-mediated humoral immunity in therapeutic success (49). On the other hand, B cells can exert pro-tumor effects through immunosuppressive mechanisms. Regulatory B cells contribute to immune evasion by expressing programmed cell death 1 ligand 1 (PD-L1) and secreting transforming growth factor-β (41). The cytokine suppresses T cell functions (50). Moreover, B cells promote tumor progression by producing fibrinogen-like protein 2, which drives M2 macrophage polarization. This factor is positively correlated with immunosuppressive mediators including IL-10, CCL5, IL-13, and vascular cell adhesion protein (VCAM) 1, thereby reinforcing an immunosuppressive TME in EC (51).

## 3 EC and TLS

### 3.1 Formation of TLS

TLS formation is co-regulated by a variety of chemokines, cytokines and adhesion molecules. This process is particularly pronounced within the framework of persistent inflammation. It is similar to what is observed in tumors, autoimmune diseases, viral infections, and chronic diseases typified by suppurative sweating inflammation (52). From the standpoint of molecular mechanisms, the initiation of TLS usually begins with local inflammation. The production of proinflammatory cytokines and chemokines creates the conditions for the *de novo* generation of lymphoid structures (20). Cellular activators encompass a diverse array of both lymphoid and non-lymphoid cells, including cancer-associated fibroblasts frequently present in tumors. These fibroblasts express adhesion molecules like vascular cell adhesion molecule-1 (VCAM-1) and intercellular cell adhesion molecule-1 (ICAM-1), and secrete pivotal chemokines such as CXCL13, chemokine ligand (CCL) 19, and CCL21. These molecules play a crucial role in recruiting lymphoid tissue-inducing (LTi) cells (53, 54). LTi cells play a key role in the formation of secondary lymphoid structures and TLSs by expressing lymphotoxin α1β2 (LTα1β2). This interacts with lymphotoxin β receptors (LTβR) on stromal cells, further promoting the expression of adhesion molecules and chemokines (55). Recent research has increasingly underscored the critical contribution of Tfh cells in driving the generation of TLS across various tumor types including EC. For instance, Tfh cells enhance the immune response to tumors by secreting IL-21. They also express inducible synergistic co-stimulation molecules (ICOS) and CD40L, which directly interact with B cells (39). In specific microenvironments, programmed cell death protein 1 (PD-1) + CXC-chemokine receptor (CXCR) 5-CD4+ Th -CXCL13 cells release CXCL13. This facilitates the activation and mobilization of B cells to TLS. It also promotes their differentiation via IL-21 and CD84, enhancing the anti-tumor immunity (40).

**Figure 1:**
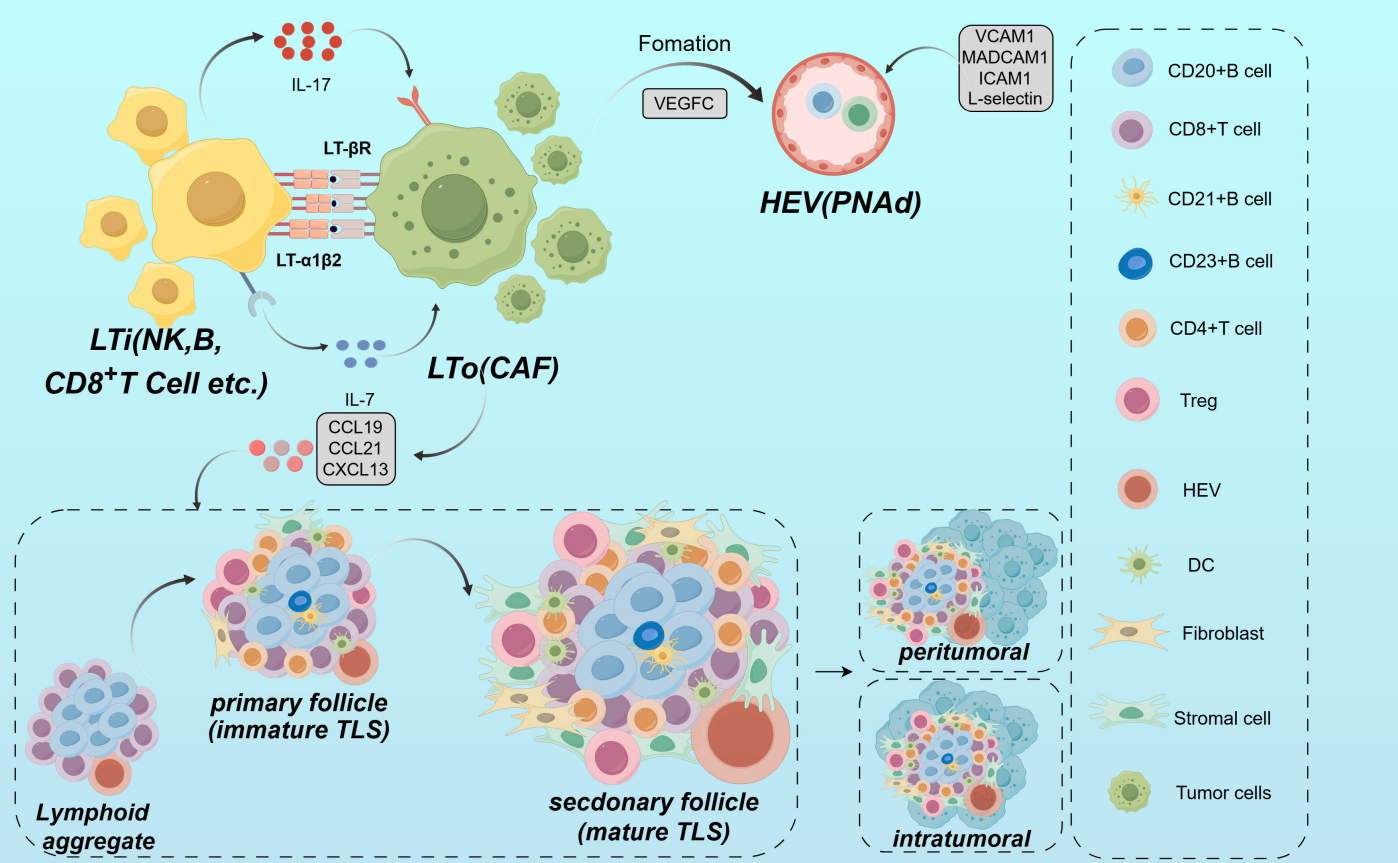
Mechanism of TLS formation. TLS is formed under local inflammation by cancer-associated fibroblasts and LTi through the interaction of adhesion molecules and chemokines (e.g., CXCL13, CCL19, CCL21). LTi cells express LTα1β2, which activates LTβR on stromal cells and promotes the organization of TLS. HEVs direct lymphocytes into the TLS via PNAd and L-selectin. The TLS contains defined regions for T cells and B cells within its structure, with the B cell region forming the GC, and the T-cell compartment comprising CD4+ T cells, CD8+ T cells.

From the perspective of structural characteristics, TLSs exhibit structural similarities to secondary lymphoid organs (SLOs), with structures such as B cells, T cells, DCs, macrophages, GCs and high endothelial microvessels (HEVs) (56). The main difference between TLSs and SLOs lies in the timing and location of formation. SLOs form during embryonic development, whereas TLSs develop *de novo* at the site of chronic inflammation or infection. In addition, TLSs do not have membranes. Rapid infiltration of lymphocytes and cytokines from the adjacent inflammatory milieu establishes a setting that is notably responsive to local antigens, including self-antigens (57). In ESCC, these differences allow TLSs to exhibit unique adaptations in the fight against local antigens. This interaction enhances tissue formation in TLS by supporting the regionalized distribution of T and B cells in ESCC (42). Crucially, the development of a functional blood supply within the TLS plays a key role. The structure of these specialized microvessels allows a large number of lymphocytes to enter the TLS from the bloodstream (58). HEVs express peripheral node addressin (PNAd), which interacts with L-selectin on lymphocytes and directs them to enter the TLS (59). The establishment of the HEVs is thought to be mediated by the localized production of LTi and stromal cells induced by vascular endothelial growth factor (VEGF). VEGF also stimulates the formation of the necessary lymphatic drainage network (59, 60). From the viewpoint of cellular composition and functional organization, TLS in EC exhibits a highly compartmentalized spatial structure. The B cell zone is centrally composed of B cells. These B cells typically form dense GCs around follicular dendritic cells (FDCs). Within this B cell zone, FDCs support GC development by providing both antigen and survival signals. They capture antigen-antibody complexes via surface Fc receptors and complement receptors. This process establishes a long-term antigen reservoir that delivers sustained signals for B cell affinity maturation (45). GC, as the site for somatic hypermutation and class-switch recombination in B cells, fosters the generation of antibody diversity and the production of high affinity anti-tumor antibodies (42, 61). In ESCC, the T cell region of TLS consists mainly of CD4+ T cells and CD8+ T cells. CD4+ T cells are densely aggregated at the peripheral region of the B cell zone. In contrast, CD8+ T cells are more diffusely distributed, infiltrating the outer edges of the TLS (62). Within the TLS, CD4+ T cells can be further classified into various subsets. Tregs, by secreting immunosuppressive factors like transforming growth factor-β, suppress excessive immune responses and are essential for sustaining immune tolerance and mitigating the risk of autoimmune responses in ESCC (41). In contrast, other CD4+ T cells, aside from Tregs, as well as CD8+ T cells, demonstrate anti-tumor effects during the anti-tumor immune response (33, 41, 63). From the overall effect, CD4+ T cells and CD8+ T cells collaboratively exert a crucial anti-tumor effect within the TLS. In the supporting cell network, DCs are positioned at the T-B cell boundary and play a critical role in antigen presentation, creating a functional interface that bridges innate immunity with adaptive immunity (36, 64). Macrophages are widely distributed across all layers of the TLS. They are tasked with clearing non-functional lymphocytes and apoptotic cell debris. Additionally, they preserve the homeostasis of the microenvironment and regulate the intensity of immune responses through the secretion of anti-inflammatory factors like IL-10 (30, 65). HEVs in the TLS extend throughout the peripheral region, specifically facilitating the homing of peripheral blood lymphocytes to the TLS (58–60). The formation of HEVs is regulated by VEGF. VEGF not only promotes the local generation of LTi and stromal cells but also stimulates the development of the lymphatic network. This ensures the exchange of materials between the TLS and its external environment (59, 60) (**Figure 1**). From a dynamic regulatory perspective, the chemokines and cytokines in TLS are rich and diverse, reflecting their dynamic and reactive nature. Key chemokines include CXCL13, CCL19, and CCL21. These chemokines specifically attract B cells and T cells through their receptors CXCR5 and chemoattractant cytokine ligand (CCR7). They promote not only the entry of immune cells into the TLS but also their organization in functional regions (52, 55, 66). For example, CD4+ T cells deficient in special AT-rich sequence-binding protein-1 promote TLS assembly by upregulating ICOS expression (41), indicating the potential role of transcriptional regulation in TLS formation (**Table 1**).The development of TLS is a multifaceted and dynamic phenomenon shaped by various factors, such as inflammatory cytokines, chemokines, adhesion molecules and the local vascular architecture. These structures are crucial in mediating the immune response to chronic inflammation and cancer as sites of antigen presentation, lymphocyte activation and clonal expansion. Understanding the mechanisms of TLS formation not only sheds light on fundamental immunological processes but also opens avenues for new therapeutic strategies against cancers like EC.

**Table 1:** Cytokines and chemokines play a crucial role in the formation of TLS.

Cell factor type	Cell factor of origin	Functional description
CXCL13	FDC, stromal cells	Attracts CXCR5-expressing B cells and T cells to the B cell zone and promotes the formation of GC
CCL19	stromal cells	Attracts CCR7-expressing B cells, T cells and DCs to TLS and promotes immune cell interactions
CCL21	stromal cells	Attracts immune cells expressing CCR7 and supports lymphocyte migration and structural organization of the TLS
TNF-α	Macrophages	Promotes inflammatory responses, enhances intercellular signaling, and supports TLS maturation and functionalization
LTα1β2	LTi cells	Interacts with LTβR and activates stromal cells, promotes the levels of adhesion molecules and chemokines, and contributes to the formation of TLS
VCAM-1	Stromal cells, cancer-associated fibroblasts	Acts as an adhesion molecule to promote immune cell adhesion and cell-cell interactions, and supports cellular localization and interactions within the TLS
ICAM-1	Stromal cells, cancer-associated fibroblasts	Supports intercellular adhesion and migration of immune cells

### 3.2 Detection methods and immune modulators for TLS

**Figure 2:**
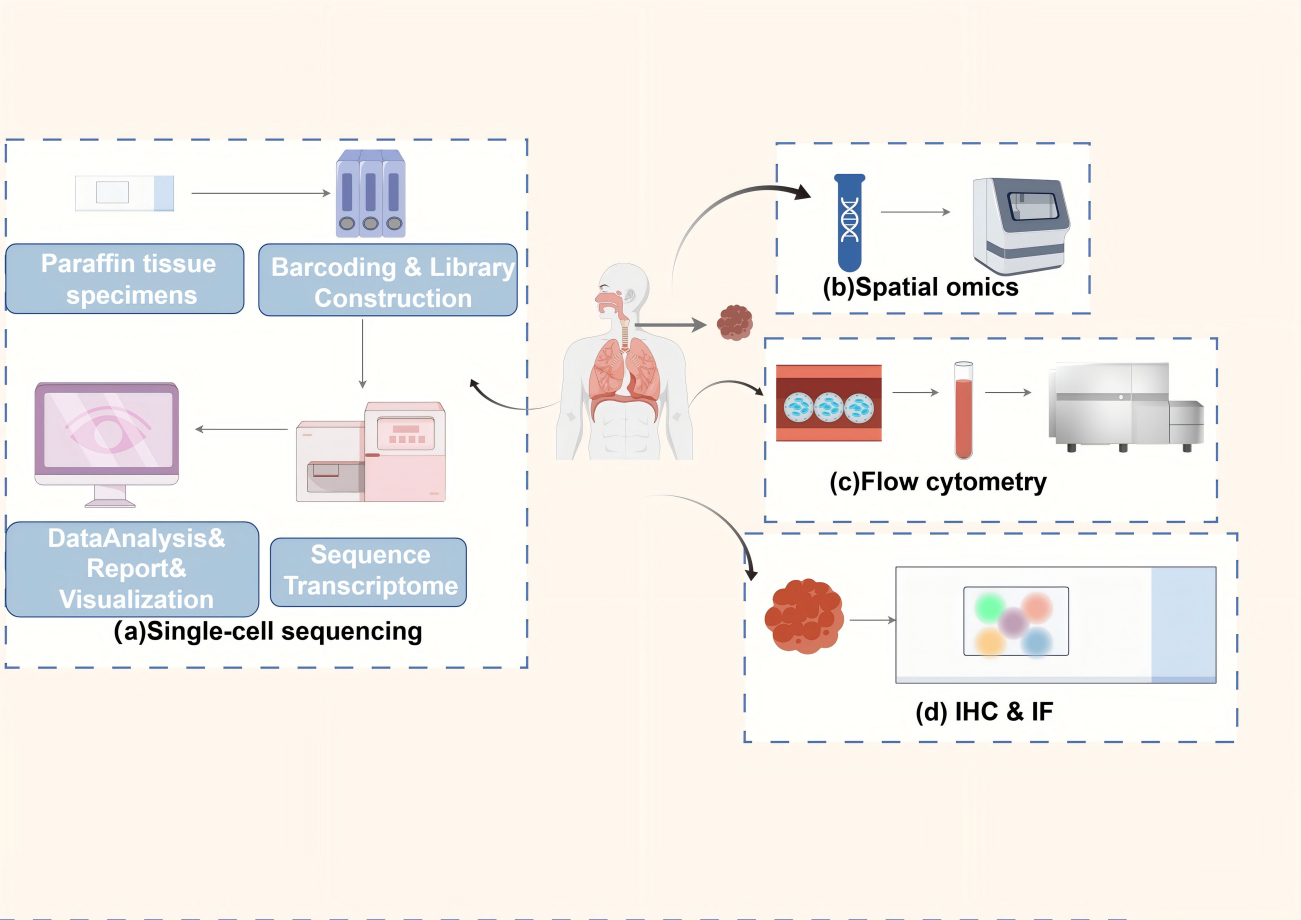
Primary methods for detecting TLSs in ESCC. The main methods for detecting inflammatory cell infiltration and immune cell subpopulations, as well as TLS in the TME of EC. **(a)** single-cell sequencing: cells are extracted from paraffin-embedded tissue samples, barcoded and library constructed, and subsequently subjected to transcriptome sequencing and transcriptomic data are reported on a single-cell level through data analysis and visualization; **(b)** spatial genomics: tissue slices are assays to obtain information on the spatial distribution of different cells in the tumor and its microenvironment; **(c)** flow cytometry: cell sorting and flow cytometric analysis of tumor tissue samples to identify and quantify the proportions of different immune cell subpopulations in TLSs in the tumor tissue; **(d)** Immunohistochemistry and immunofluorescence techniques: specific antibodies are used to label immune cells in TLSs, and the labeled immune populations (e.g., CD20) are quantified by a digital pathology image analysis system to provide information on their type, number and location.

Histological evaluation of TLS in EC needs to consider the infiltration of inflammatory cells in the esophageal TME, such as lymphocytes, plasma cells, and macrophages. It also requires evaluating the internal immune cell types and densities, such as T cells, B cells, and DCs, which affect tumor growth, metastasis, and response to therapy in EC (67). In addition, the assessment of tissue architecture and degree of maturation in TLS is performed. This, along with the number of infiltrating immune cells and their spatial distribution, provides a comprehensive understanding of the pathologic features and clinical manifestations (68). At present, the predominant approaches involve identifying TLS and its various immune subpopulations in EC tissue specimens by immunohistochemistry and flow cytometry. IHC uses specific antibodies to label immune cells and quantifies the labeled populations, such as CD20, CD21, CD4, CD8, and Pan-CK. A digital pathology image analysis system provides information on the type, quantity, and spatial arrangement of TLS in EC, including their distribution within and surrounding the tumor (69, 70). Flow cytometry allows the analysis of individual cells to identify the subpopulations of immune cells within them and quantify their proportion in the TLS of EC tissues (34). In addition to these approaches, several other emerging technologies, such as single-cell sequencing, spatial transcriptomics and spatial proteomics analysis, are being applied to scientific research, precision diagnosis, and treatment of TLS in EC (33, 44, 71) (**Figure 2**). The detection of multiple ectopic lymphoid aggregates featuring both CD21+ B lymphocytes and CD3+ T lymphocytes is essential in the early stages of TLS, whereas mature TLS exhibits structures with GCs and lymphoid follicles (CD21, CD23). Finally, by measuring the total areas of the EC and the areas of tumor-associated lymph nodes (TLOs) in different locations (intratumoral and peritumoral), the proportion of total TLSs relative to the total EC tissues can be determined (69). At present, the majority of techniques for assessing TLS rely on the image segmentation of hematoxylin and eosin-stained sections (72, 73). This approach necessitates significant training in image processing and substantial computational resources to yield more precise data. While research on the evaluation techniques of TLS in EC is limited, these methodologies provide valuable insights for identifying other tumors. To illustrate, Chen et al. used deep learning and inForm software for TLS segmentation of regions of interest in multiple immunohistochemical staining (mIHC) whole slide images, which improved the ability to identify TLSs (70). Li et al. employed machine learning and the residual neural network 18 convolutional neural network to delineate tumors from normal tissues, while also utilizing region-based convolutional neural networks for the segmentation and classification of individual cell nuclei (72). A machine learning model was developed to identify and classify TLS. It also quantitatively assessed TLS using lymphocyte density maps. The model established correlations with patient prognosis and gene expression profiles (72). Wen et al. used deep learning and the Vectra Polaris system for automated quantitative pathology imaging as well as the convolutional neural network INSTERATION-RESNET-V213 to build a TLS classifier, which was successful in identifying and distinguishing mature and immature TLSs (73). Hu et al. used imaging mass spectrometry cytometry combined with spatial proteomics analysis. They showed that the core of the TLS consists of densely packed B cells and CD8+ T cells. These cells synergistically enhance the anti-tumor immune response through proximity (5-20 μm) interactions. In the post-treatment good-response group, the number of CD8+ T cells and Ki-67+ proliferating B cells within the TLS was significantly increased (71). This immune activation effect was also reflected outside the TLS, as activated memory CD8+ T cells (CD45RO+) formed a supportive immune environment within 10-20 µm around the TLS (71). In the poor response group, the TLS was surrounded by suppressor cells. These included PD-L1+ macrophages and Foxp3+ Tregs. These cells attenuated the immune effect by secreting immunosuppressive molecules or forming a fibrotic microenvironment (e.g., high collagen I expression). This spatial relationship between fibrotic areas and immunosuppression limits T cell infiltration. It also exacerbates immunotherapy resistance by enhancing the inflammatory activity of S100 calcium-binding protein A9+ macrophages (71). Nakamura et al. utilized single-cell RNA sequencing to discover that TLS in ESCC significantly enhances the anti-tumor immune response by promoting CD8+ T cell cytotoxicity, antigen presentation by DCs, and B cell maturation (33). Specifically, CD8+ T cells in TLS+ tumors exhibited upregulation of cytotoxic genes such as *GZMK* and *PRF1*, highlighting a notable enhancement in cytotoxic characteristics (33). Additionally, DCs in TLS showed increased expression of major histocompatibility complex (MHC) class II molecules and co-stimulatory molecules, thereby exhibiting stronger antigen presentation functions (33). Furthermore, semaphorin 4D, a critical ligand secreted by Tfh cells, modulates the antigen presentation function of DCs. Lastly, Tfh cells upregulate ICOS and PD-1 within TLS, which further enhances Tfh cell-B cell interactions and facilitates the progression of the GC response (33). Recently, Huang et al. utilized single-cell RNA sequencing to uncover the multidimensional regulatory network of TLS in ESCC (34). CCL19/CCL21-CCR7, CXCL13-CXCR5, and LTα1β2-LTβR represent the core chemotactic and activation pathways of TLS in EC. Regarding chemokine-receptor interactions, stromal cells within TLS secrete CCL19 and CCL21. These molecules bind to CCR7 and attract T cells, B cells, and DCs. This promotes immune cell aggregation. Concurrently, CXCL13, produced by FDCs and stromal cells, recruits B cells and Tfh cells to the B cell zone via CXCR5, thereby supporting the formation of the GC. In terms of key signaling pathways, LTi cells activate stromal cells by secreting LTα1β2. This leads to the upregulation of VCAM-1 and ICAM-1 expression, driving the maturation of TLS. Additionally, macrophages secrete tumor necrosis factor (TNF) -α, enhancing stromal cell signaling and promoting the structural stability of TLS (34). Notably, immune suppressive mechanisms are also present in TLS of EC. On the one hand, Tregs inhibit TLS function through cytotoxic T- CTLA4. on the other hand, in TLS of EC, CD20+IgD-CD27- double-negative B cells fail to effectively activate T cells due to the absence of CD27, however, such double-negative B cells represent a very small proportion within TLS (34). Liu et al. employed single-cell sequencing and spatial transcriptomics to identify the dynamic evolution and functional activation mechanisms of TLS: mature TLS is marked by the CD23+ FDC network, with a significant increase in the proportion of HLA-A+ cells. The primary components consist of CXCL13+ exhausted CD8+ T cells and CD4+ CXCL13+ T cells. Specifically, CXCL13+ exhausted CD8+ T cells upregulate antigen presentation-associated genes such as *HLA-A*, *HLA-B*, *TAPBP*, and cytotoxic molecules IFN-γand GZMB, facilitating functional reactivation. While CD4+ CXCL13+ T cells enhance B cell activation and support TLS maintenance by expressing co-stimulatory receptors tumor necrosis factor receptor superfamily (TNFRSF) 9 and tumor necrosis factor superfamily member (TNFSF) 14. B cell-rich -A+ T cells within TLS exhibit significantly elevated expression of CXCL13 and antigen presentation-related genes, reinforcing their antigen presentation function (44). The CXCL13-CXCR5, HLA-A-mediated antigen presentation, and TNFRSF9 signaling pathways are critical regulatory axes in TLS of EC. In terms of key signaling pathways and molecular interactions, CXCL13-CXCR5 drives the recruitment of lymphocytes to TLS, promoting its structural development. Concurrently, T cells within TLS enhance MHC class I antigen presentation by overexpressing HLA-A, thereby activating CD8+ T cells to initiate anti-tumor responses. In terms of co-stimulatory signals, TNFRSF9 is notably upregulated in terminally differentiated CXCL13+ exhausted CD8+ T cells, enhancing their survival and effector functions. Meanwhile, TNFSF14 facilitates the interaction between B cells and T cells, thereby supporting the GC response (44).In general, various techniques are employed in TLS research to decode its dynamic mechanisms, each method offering unique advantages. However, the limitations of these techniques must be weighed and we should choose them based on the specific research objectives (**Table 2**). Specifically, immunohistochemistry and immunofluorescence are suited for preliminary localization and phenotypic analysis. These methods preserve tissue spatial information and localize TLS markers such as CD20 and CD3. However, they have low throughput, a limited number of targets (typically ≤5 markers), and rely on subjective interpretation. This makes it difficult to capture cellular heterogeneity. Flow cytometry allows high-throughput analysis of immune cell phenotypes and functional states within TLS. However, it requires the preparation of single-cell suspensions, which compromises tissue architecture and spatial information. This prevents it from resolving the spatial characteristics of TLS. Advanced technologies such as machine learning models, single-cell sequencing, spatial transcriptomics and spatial proteomics provide a more refined and dynamic analysis for the pathological assessment of TLS in EC, and they are able to reveal the complexity of the TME in comparison to traditional methods. Machine learning, by integrating multi-omics data, can predict TLS-associated biomarkers, but its performance is heavily reliant on data quality and annotation accuracy, and it is difficult to uncover the underlying molecular mechanisms. Single-cell sequencing provides transcriptomic maps at single-cell resolution. It can identify rare cell subpopulations within TLS, such as Tfh or B cell clones. However, it is costly and loses spatial localization, requiring *in situ* validation. Spatial transcriptomics preserves gene expression and tissue localization using spatial barcodes, allowing for the mapping of immune cell distribution gradients within TLS. However, its resolution makes it challenging to distinguish individual cells, and it cannot directly detect protein activity. Spatial proteomics directly quantifies the spatial expression of dozens of proteins, shedding light on intercellular interactions and signaling pathways. However, it relies on antibody quality, has limited throughput due to imaging techniques, and cannot simultaneously capture transcriptomic data. Characterization of TLS and anti-tumor immunity.

**Table 2:** A comparison of methods for TLS detection.

Methods	Advantages	Disadvantages
Immunohistochemistry /Immunofluorescence	Preserves spatial structure and localizes markers (e.g., CD20 and CD3).	Low throughpu, subjective quantification, inability to resolve single-cell heterogeneity.
Flow cytometry	High-throughput phenotypic analysis, detects cell function (e.g., cytokine secretion).	Disrupts spatial structure, unable to associate tissue microenvironment with spatial features.
Machine learning	Identifies complex biomarkers and predicts the association between TLS and prognosis.	Relies on high-quality labeled data, low model interpretability.
Single-cell sequencing	Single-cell resolution, reveals cellular subpopulation heterogeneity within TLS (e.g., Tfh and B cell clones).	High cost, loss of spatial information, requires *in situ* validation,
Spatial transcriptomics	Preserves gene expression and spatial localization, maps immune cell distribution gradients.	Limited resolutio, unable to detect protein activity.
Spatial proteomics	Quantifies multiple proteins in space, reveals cellular interactions and signaling pathway dynamics.	High antibody dependence, limited throughput, difficult to simultaneously capture transcriptomic data.

TLSs are critical environments for supporting plasma cells in antibody secretion for antigen clearance, regulating T cell activation, and facilitating the maturation and differentiation of B cells (74). Early-stage TLSs demonstrate diverse levels of B and T cell clustering, yet are devoid of FDCs or more complex formations such as GC and HEV. These structures can be located in different areas of the primary tumor, including the tumor’s periphery, its core, and surrounding epithelial tissues (75). In EC patients responding to immune checkpoint therapy, there is a notable increase in the density of B cells, especially converted memory B cells. Specific T cell subsets, such as CD8+ T cells, are also increased (76). CD20+ B cells are more abundant in the tumors of responders compared to non-responders. These cells co-localize with CD8+ T lymphocytes in TLS of EC, enhancing the anti-tumor immune response. Additionally, CD8+ T cells infiltrate the follicular center border and adjacent lymphocyte clusters, facilitating TLS formation in EC (76). In EC, TLSs enhance the identification and elimination of tumor cells by immune cells through direct contact with tumor cells. This positional advantage allows TLSs to effectively organize and mobilize the immune response against tumor cell invasion and the spread of EC (36). Mature TLSs in EC give rise to GCs, FDCs, HEVs and various other components (36). Within the GCs, B cells of EC exhibit a remarkable rate of proliferation and are activated to maturity Ki-67+, enhancing anti-tumor immune responses (36). B cells demonstrate significant somatic hypermutation and serve as crucial antigen-presenting cells, producing anti-tumor antibodies and enhancing anti-tumor humoral immunity (36). In mature TLS+ tumors, CD4+ and CD8+ T cells as well as memory B cells were more abundant (33). Foxp3+ Tregs are generally seen as facilitators of tumor advancement. In the peripheral regions of tumors, CD8+ TLSs are both depleted and functionally suppressed, alongside Foxp3+ Tregs. These cells are characterized by elevated PD-1 expression (77). In EC, CD21+ FDCs serve as antigen-presenting cells, modulating T cell development through IL-7 secretion. This regulates B cell recruitment and differentiation in GCs (78). CD68+ macrophages and CD163+ macrophages are involved in maintaining the immunosuppressive microenvironment of early TLS in EC (79). Conversely, a separate investigation revealed that mature TLSs in EC exhibit the expression of CD68+ and CD163+ macrophages, their immunosuppressive effects may be counteracted by the surrounding activated immune environment (30). Mature lysosome-associated membrane glycoprotein+ DCs exhibit a robust antigen-presenting capability, while B cells can facilitate the maturation of these DCs (80). TLSs in EC can be categorized into three maturation stages based on their cellular composition (30, 73). However, there is currently no research or reports in EC regarding the correlation between different surrounding cells outside of TLSs and different subtypes of TLSs, such as their level of organization or maturation. Despite significant research identifying various elements that influence TLS formation within TME of EC, our comprehension of the surrounding cells that either promote or impede TLS maturation remains incomplete. Future research should focus on categorizing TME lacking TLS formation or maturation based on unique characteristics. These could be classified as ‘restrictive’ or ‘insufficient’, depending on the absence of crucial factors, such as antigens. It is noteworthy that recent studies have uncovered the potential pro-tumor functions of TLS in EC (30). In EC, TLS functions as a double-edged sword, possessing both anti-tumor and pro-tumor roles. Research has revealed that in mature TLS, the density of IgG4+ plasma cells is significantly negatively correlated with CD8+ T cells (*P* = 0.04) (30). This is mediated by the low-affinity interaction between the Fc region of IgG4 and Fcγ receptors. This interaction directly inhibits antitumor responses, including antibody-dependent cell-mediated cytotoxicity, antibody-dependent phagocytosis, and complement-dependent cytotoxicity (30). Moreover, IgG4 interferes with the function of IgG1 via Fc-Fc interaction (30). In comparison to healthy controls, cancer patients exhibit significantly higher levels of IL-10 and IgG4 in their serum, with a positive correlation between the two (*P* < 0.01) (30). IL-10, secreted by Tregs, CD163+ M2 macrophages, and CD206+ M2 macrophages, drives the conversion of B cells into IgG4+ plasma cells, thus establishing an immunosuppressive loop (30). Additionally, *in vitro* experiments have demonstrated that IgG4 can directly induce M2 macrophage polarization, as evidenced by the upregulation of CD163 and the downregulation of IL-1β and inducible nitric oxide synthase (30). In conclusion, IgG4 plays a crucial role in supporting EC progression by inhibiting CD8+ T cell function and cooperating with IL-10. These actions drive M2 macrophage polarization and B cell class switching within TLS, thereby providing essential microenvironmental support.

### 3.3 Prognostic value of TLS

**Figure 3:**
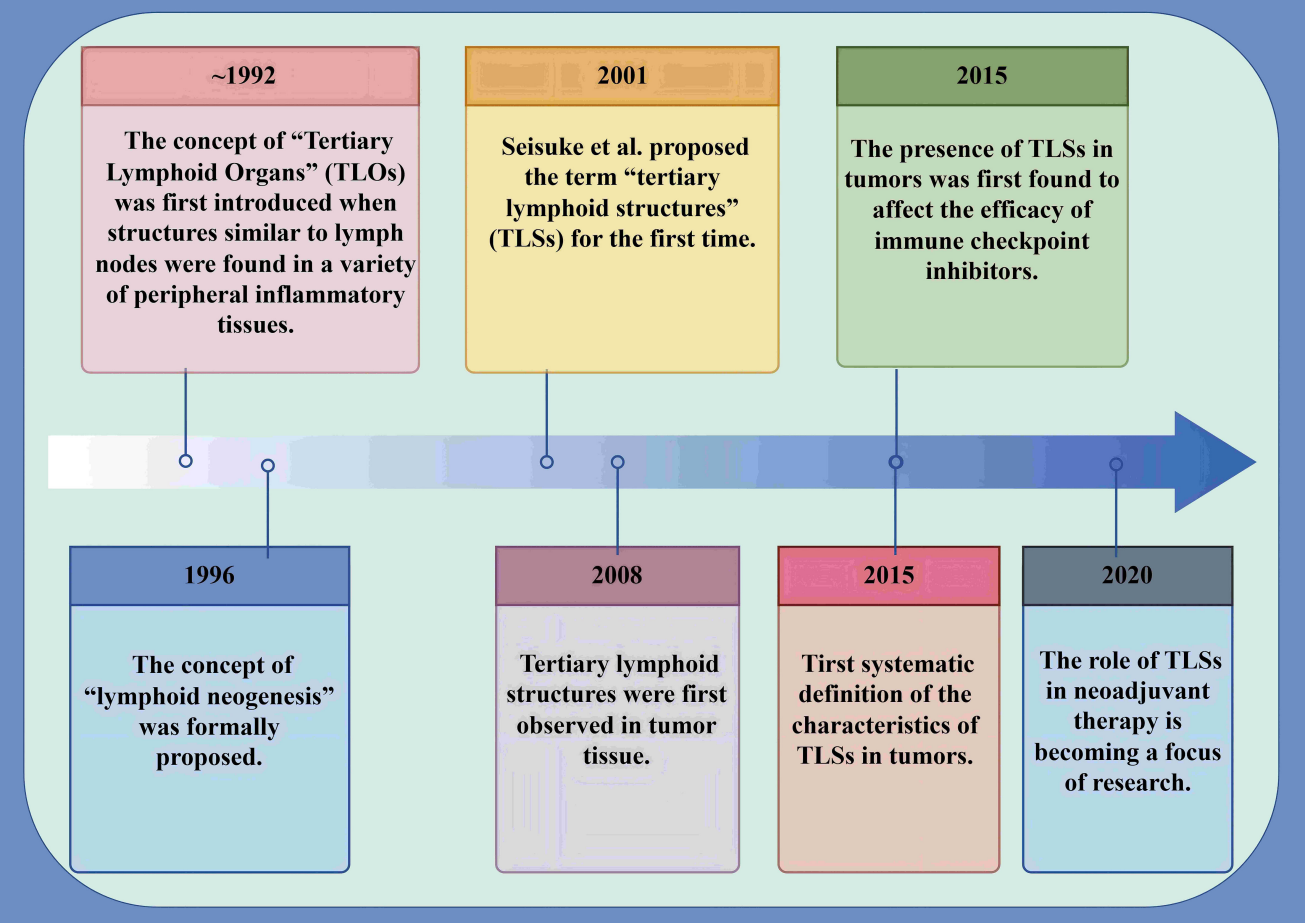
Milestones in the identification and advancement of TLS within tumors. Milestones underscore the relevance of TLS within tumors. This chart encapsulates the definition of TLS in tumor contexts, outlines the trajectory of their discovery, and highlights recent research advancements. TLSs are abnormal clusters of lymphocytes that emerge in non-lymphoid tissues during pathological states. The foundational concept was first introduced in 1992, with the designation “TLS” being established in 2001. Over the last ten years, TLS has gradually become a focus of research and studied extensively and in-depth.

**Figure 4:**
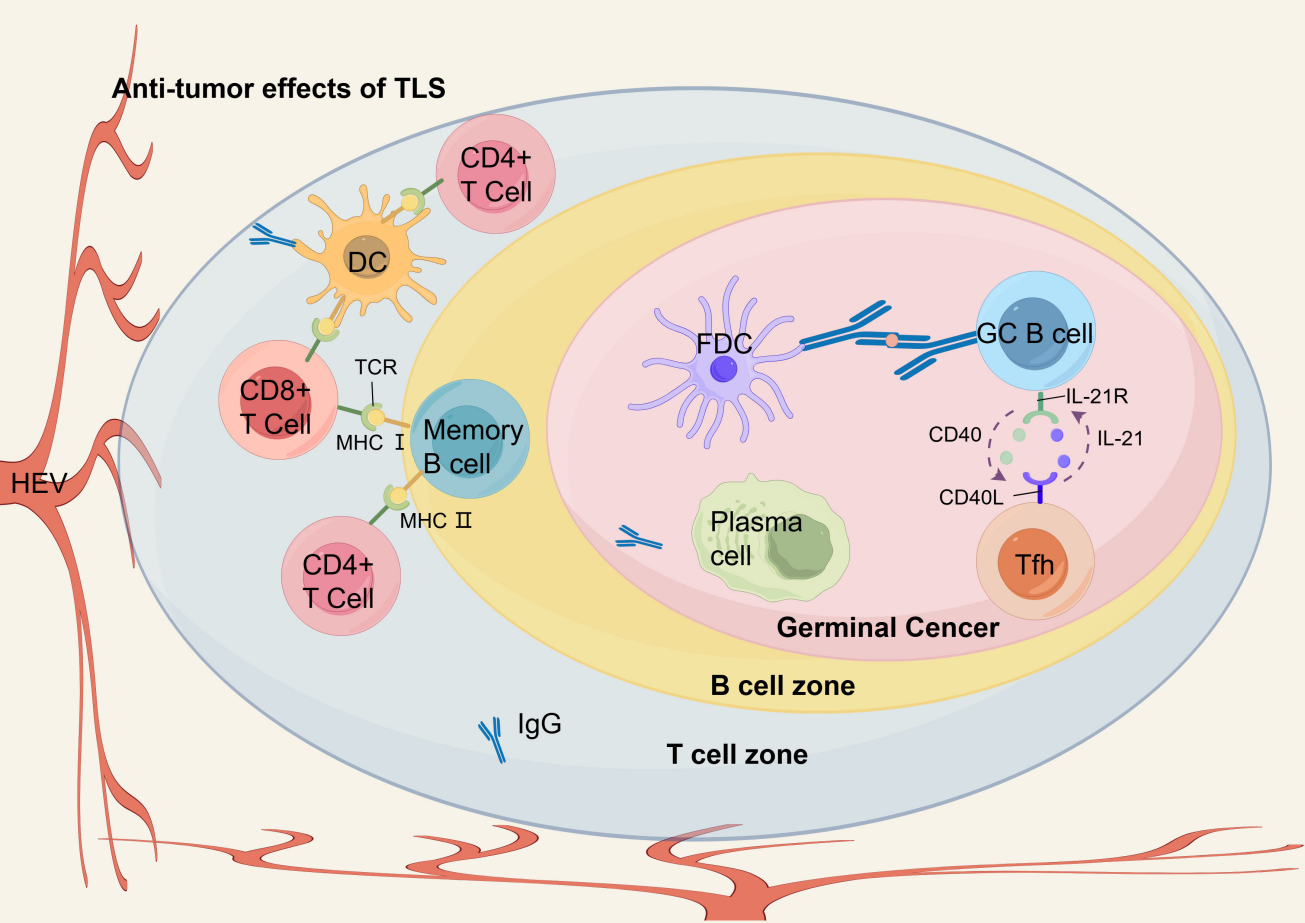
The anti-tumor immune response of TLS in EC. Within the GC of TLS, GC B cells undergo differentiation into memory B cells and plasma cells upon receiving antigen delivery from FDCs and engaging with Tfh cells through CD40 and IL-21 interactions. In the T cell zone, differentiated B cells and DCs present antigen peptides to CD8+ T cells and CD4+ T cells via MHC I and MHC II pathways, respectively, thereby facilitating the infiltration of effector T cells into the TME. Plasma cells within TLS produce anti-tumor IgG. HEVs in the peripheral areas recruit peripheral blood lymphocytes into TLS, thereby ensuring a sustained supply of immune cells that support anti-tumor immunity.

In EC, TLSs are populated with TILs, and their distinctive architectural characteristics facilitate direct interactions with tumors, enabling a swift and potent anti-tumor immune response (36). Numerous studies have indicated the presence of intra-tumoral TLSs in bladder cancer, lung cancer and head and neck squamous carcinoma tissues contributes to the improvement of patient prognosis (24, 80, 81). On the one hand, TLS acts as a general prognostic marker for EC. TLS is often found at tumor invasion margins and is linked to better prognosis in EC patients, with higher TLS density and maturation associated with prolonged survival (82). An investigation was carried out to explore the correlation between the presence of TLS and prognosis in EC, with Jing et al. assessing TLS in a cohort of 185 patients following surgical resection (45). The investigation revealed that the presence of TLS in EC serves as an independent prognostic indicator, with patients exhibiting TLS positivity experiencing extended disease-free survival (DFS) and OS in comparison to their TLS-negative counterparts (*p* < 0.01) (45). In addition, the study emphasized that an increased accumulation of CD20+ B cells of TLS was associated with improved survival outcomes, demonstrating its potential role in enhancing anti-tumor immunity in EC (45). Bonamino et al. reported a comparable observation in a cohort of 236 patients diagnosed with ESCC (83). This suggests that the density and maturity of TLS may serve as biomarkers of prognosis in patients with EC. Doki et al. found that in 316 patients with ESCC, high-density and mature peritumoral TLS were linked to lower tumor stage (*p* < 0.0001) and absence of lymphatic vessel or vascular invasion (*p* = 0.0001). They were also associated with better serum nutritional parameters (e.g., neutrophil count; *p* < 0.0001) and long-term survival (mPFS 160d vs 52d, *p* = 0.004) (83). In EC, Nakamura et al. found that in a sample of 180 patients evaluated pathologically, cases with mature TLSs showed a significant correlation between RFS (*p* < 0.01) and OS (*p* < 0.0002), with better outcomes compared to cases with immature TLSs or cases without TLS (33). Furthermore, single-cell RNA sequencing analysis of 14 EC samples demonstrated that CD8+ T cells within TLS+ tumors displayed increased expression of cytotoxic markers, while the antigen presentation capacity of DCs was enhanced in these tumors (33). In EC, Gao et al. found that in 316 EC patients with high-density and mature TLSs compared to those with low-density TLSs, OS was prolonged (median 160d vs 52d, *p* < 0.005) and had lower tumor stage and less lymphovascular or vascular invasion (84). The density and maturation of TLS serve as significant biomarkers for forecasting the survival of patients with EC and their response to anti-PD-1 antibody therapy. In EC, Jin et al. demonstrated that among 593 patients, an infiltrative tumor growth pattern characterized by IFN-γ was linked to reduced levels of TILs or the absence of TLS. Moreover, this growth pattern was further correlated with extensive tumor invasion, poor histological differentiation, pronounced lymphovascular invasion and increased lymph node metastasis in EC(*p* < 0.001) (85). Analysis of OS revealed that IFN-γ levels and low-grade TILs in EC serve as independent adverse prognostic indicators (IFN-γ: *p* < 0.02; TILs: *p* < 0.003) (85). Furthermore, a novel risk stratification model has been developed. This model, informed by the status of inflammatory factors and TILs, could predict the prognosis of patient survival in EC (85) (**Table 3**). Since the formation and presence of TLS in EC are linked to favorableprognostic outcomes for patients, it shows its potential value in tumor therapy. A recent study has pointed out that inducing the formation of TLS may represent a novel avenue for advancing tumor immunotherapy (27) (**Figure 3**).On the other hand, TLS serves as a predictive marker for immunotherapy in EC. In a cohort of 34 EC patients treated with anti-PD-1 antibodies, TLS density was determined as a predictive factor for immunotherapy response (83). Doki et al. discovered that high density TLS was associated with improved clinical response (*P* = 0.007) and prolonged survival (*P* = 0.004) compared to the low density TLS group (83). In immunotherapy, especially PD-1 blockade therapy (ICB), the expression of cytotoxic genes *IFNG* and *GZMB* is upregulated in EC, along with increased expression of antigen presentation molecules HLA-A and co-stimulatory receptor TNFRSF9 (44). Moreover, the presence of B cell-rich HLA-A-positive TLSs in ESCC is associated with better therapeutic response, and B cells promote antibody-mediated tumor recognition and cytokine secretion through their activation in TLSs of ESCC, thereby improving the efficacy of ICB (44). Positive regulatory mechanisms include the clustering of CD8+ T cells and B cells in TLS, which amplifies their tumor-killing and antigen-presentation capabilities. The activation of NK cells also synergizes with PD-1 inhibitors to enhance the innate immune response (44). Conversely, negative regulatory mechanisms comprise Collagen I+ fibroblasts remodeling the extracellular matrix. This limits immune cell infiltration and secretes immune suppressive factors. PD-L1+ macrophages inhibit T cell activity via the PD-1-PD-L1 axis, thereby diminishing the effectiveness of immunotherapy. And S100 calcium binding protein A9+ macrophages in the fibrotic zones surrounding TLS suppress T cell functionality through inflammatory processes and metabolic reprogramming (44). T cell factor (TCF) -1+CD39+PD-1+CD8+ T cells (CD39+ Tpex cells) are predominantly found in TLS, characterized by high proliferative activity and stemness maintenance traits, and they act as critical precursor cells for ICB responses (77). Tumors with TLS exhibit markedly higher response rates to ICB (*P* < 0.05), and the density of CD39+ Tpex cells in TLS correlates positively with the clinical benefits of ICB (*P* < 0.001). Additionally, the density of CD39+ Tpex cells in TLS is significantly greater than in the surrounding stroma and tumor parenchyma (*P* = 0.021), and their proliferative activity continues to rise after ICB. This highlights TLS as a potential predictive marker for ICB response in ESCC (77). TCF-1-CD39+PD-1+CD8+ T cells (CD39+ dTex cells) are a differentiated subset of exhausted T cells. They are primarily found in the tumor parenchyma and are linked to direct anti-tumor activity (77). TLS facilitates the recruitment and expansion of CD3+ Tpex cells. Upon loss of TCF-1, these cells migrate from TLS to the tumor stroma and parenchyma, where they differentiate into CD39+ dTex cells (77). TLS not only serves as an independent prognostic factor for EC but also functions as a critical biomarker for predicting immune therapy response. The dual functional characteristic underscores the potential clinical value of TLS in immunotherapy for EC and provides a theoretical foundation for optimizing treatment stratification and personalized immune therapeutic strategies (**Figure 4**).

**Table 3:** Comparison of the prognostic relevance of TLS in EC.

Type of cancer	Sample size	Key findings	Statistical significance
ESCC	185	TLS positivity prolonged DFS and OS	*p* < 0.01
ESCC	316	High density and mature TLSs are associated with lower tumor stage	*p* < 0.0001
ESCC	180	Mature TLSs are associated with better RFS and OS	*p* < 0.01, *p* < 0.0002
ESCC	316	High density and mature TLSs are associated with prolonged OS	*p* < 0.005
ESCC	593	TLSs and low-grade TILs are poor prognostic factors	*p* < 0.003

## 4 Limitations and future directions

### 4.1 Lack of basic and multicenter forward-looking research

At present, investigations into TLS in EC seem to be less advanced compared to studies on other malignancies, such as pancreatic or colorectal cancers, especially in the elucidation of molecular mechanisms, studies on induction mechanisms and multicenter prospective clinical studies. Only a few single-center prospective clinical studies that are concerned with TLS in EC have been conducted. For instance, a single-center phase 2 study by Zhou et al. evaluated EC patients treated with tislelizumab plus chemotherapy. Increased TLS density was strongly associated with improved clinical outcomes, including OS and DFS. Patients achieving a pCR exhibited elevated concentrations of T lymphocytes and B lymphocytes within the TLS (86). Another single-center phase 2 trial also showed that TLS in EC was significantly associated with “immune-infiltrating” TME, which correlated with improved response, better pathology and survival in patients treated with neoadjuvant camrelizumab plus chemotherapy (62). While the composition, interactions between cells and related signaling pathways of TLS have been explored extensively in various cancers, such as pancreatic or colorectal cancers, the molecular mechanisms underlying TLS remain inadequately understood in EC. Limited single-center EC clinical trial has integrated machine learning and spatial proteomics analysis to guide immunotherapy interventions (71). Proposed strategies include targeting inhibitory macrophages and fibrogenic factors at TLS peripheries or enhancing effector cell activation within TLS. Specifically, in EC, CD45RO+ CD8+ T cell density at the TLS-epithelial junction (5–15 μm) and the CD4+ T cell-to-B cell ratio predicted PD-1 inhibitor response. Conversely, elevated S100A9 expression and Treg ratios in TLS-surrounding fibrotic regions correlated with immunotherapy resistance (71). Most current studies on TLS in EC focus on descriptive pathological analysis and correlation with prognosis, and there is a lack of in-depth mechanistic studies or functional validation experiments to support the direct correlation between gene expression data and TLS function. Such as, how specific signaling pathways regulate TLS formation and function in EC are rarely reported in the literature. In addition, most current studies of TLS in EC are retrospective and single-center prospective, and while these studies can provide correlations between TLS and EC prognosis, their results are limited by the timeliness, completeness and universality of data collection. Multicenter prospective studies that collect data across geographic regions and populations can enhance the external validity of results, reduce selection bias and increase the generalizability of findings, leading to a more accurate assessment of the clinical value and mechanism of action of TLS in EC.

### 4.2 Lack of research on induction mechanisms

**Figure 5:**
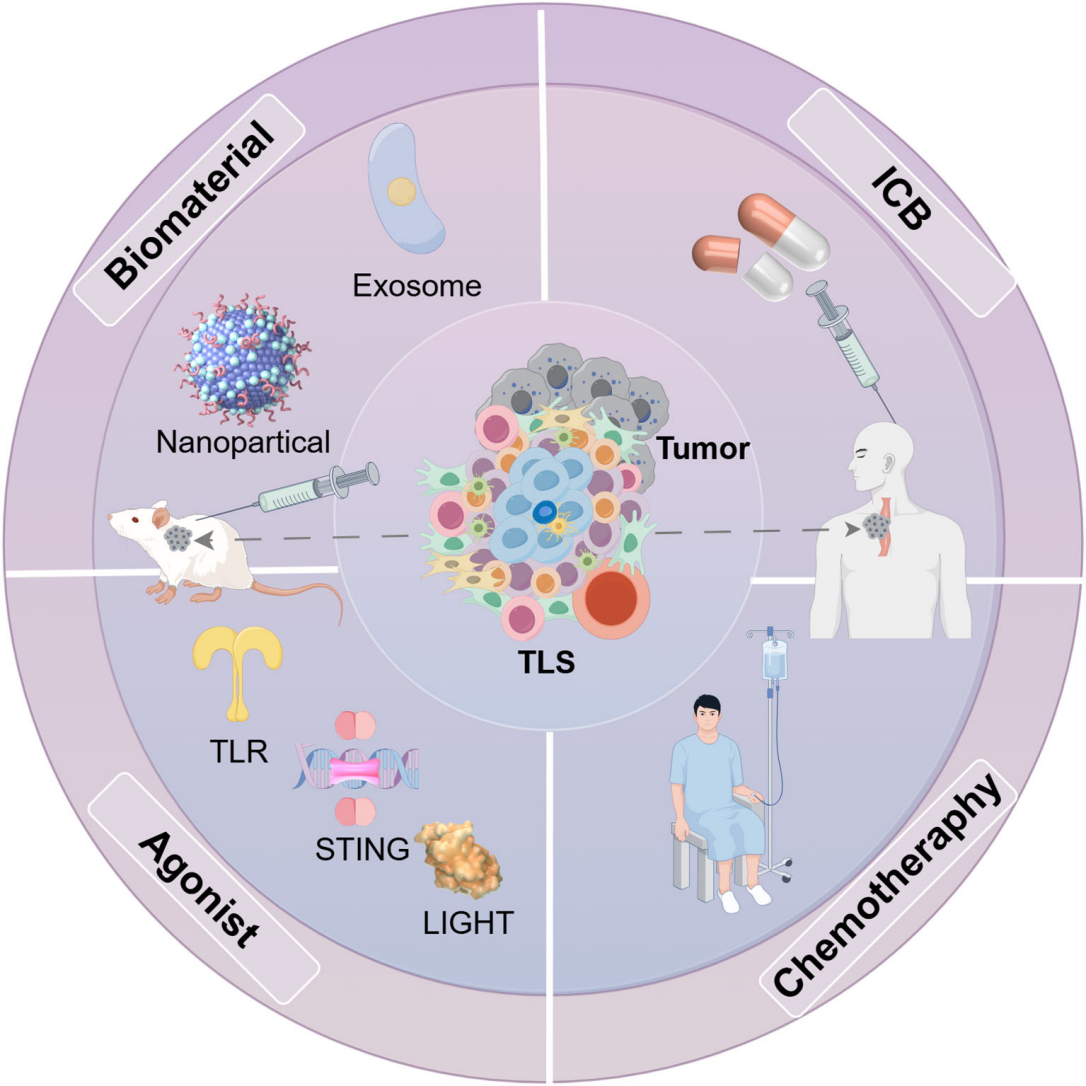
Methods for inducing TLS generation in some tumor models. Various approaches have been developed to stimulate the formation of TLS in different mouse tumor models. Key cytokines and chemokines, such as IL-13 and CCL21, that facilitate TLS development and B cell maturation can be delivered using exosomes and nanoparticles. Moreover, receptor agonists play a role in enhancing TLS formation. Furthermore, integrating multiple therapeutic strategies, including immune checkpoint therapy therapy and chemotherapy, can also influence TLS production.

In colorectal cancer, Storkus et al. injected DCs expressing T-bet and secreting bioactive IL-36γ into tumors. This approach enhanced T-bet expression in DCs, induced T cell infiltration and TLS formation, and revealed a synergistic role of T-bet and IL-36γ in promoting TLS development and TME conversion to a favorable immune environment. Furthermore, the combination of TLS induction, T-bet, and IL-36γ secretion more efficiently activated effector T cells at tumor sites (87). Specifically, T-bet, as a transcription factor, is mainly expressed on CD4+ Th1 cells and Cytotoxic T cells, and its expression in the TME promotes Th1-type immune responses and activates anti-tumor immunity (88). IL-36γ is a member of the IL-1 family that activates DCs by binding to IL-36 receptors. This interaction increases surface chemokines (e.g., CCL19, CCL21), which attract more immune cells into the tumor area, thereby promoting the formation of TLS (89). In addition, IL-36γ enhances the expression of T-bet in T-bet-expressing DCs, which further amplifies the Th1 response, allowing DCs to more effectively activate T cells in the TME and promote the formation of TLS (87). Johansson et al. found that in a pancreatic neuroendocrine tumor model, the homologous to lymphotoxins (LIGHT) and vascular targeting peptide normalized the intra-tumor vascular system. This vascular remodeling facilitated the entry of numerous effector and memory T cells into the tumor via the regular vascular network, thereby promoting the development of TLS within the tumor (90) (**Figure 5**). Experimental models have investigated chemo- and immunotherapeutic strategies aimed at promoting the formation of TLS in various cancer types, such as colorectal and pancreatic cancers. However, comparable research in EC remains largely absent. This limits the possibility of using TLS as a potential therapeutic target, as it is difficult to design targeted therapeutic strategies without understanding how to effectively induce TLS formation and activation in EC.

### 4.3 Challenges for clinical application

In a mouse model of lung cancer, Onur Boyman et al. augmented the function and population of Foxp3+ Tregs by manipulating them in favor of IL-2 signaling. This was achieved using the IL-2 and anti-IL-2 complex, which effectively promotes long-term lung transplantation survival (91). Tregs are enriched in TLS formed in the transplanted lung loci, releasing anti-inflammatory cytokines and enhancing immune tolerance, maintaining the immunosuppressed state and functional survival time of transplanted lungs (91). As we can see, even if the current study reveals the potential value of TLS in EC, how to translate these findings into practical clinical applications remains a major challenge. For TLS in EC, future studies must focus on the methodologies for evaluating TLS within standard clinical practice, as well as the adjustments to treatments based on the unique characteristics of TLS. Based on the above discussion, we hereby propose recommendations for future studies on TLS in EC. First, given the lack of current TLS studies in EC in terms of underlying mechanisms, upcoming research should delve deeper into the molecular mechanisms and cellular interactions involving TLS in EC. For example, detailed resolutions of cellular heterogeneity and cellular states in TLS through the use of advanced single-cell sequencing and spatial omics technologies will help identify key cell types and signaling pathways in EC. Future studies could apply CRISPR/Cas9 or RNAi to knockout/knockdown TLS-related genes in EC. These experiments would validate gene roles in maintaining TLS structure and function. Second, considering that most studies of TLS in EC are retrospective and single-center prospective, the development of multicenter prospective studies will be key to understanding the role of TLS in EC progression. Future studies should design multicenter, large-sample prospective clinical studies to systematically collect correlation data between TLS and clinical outcomes in EC in order to construct more accurate pathology and biomarker models. We contend that these investigations will not only yield more robust evidence regarding the association between TLS presence and the prognosis of EC but also assess the predictive value of TLS characteristics on treatment response. In addition, combining immunomodulators with traditional therapies (e.g., chemotherapy, radiotherapy) or emerging immunotherapeutic strategies (e.g., immune checkpoint inhibitors) and exploring their synergistic effects in inducing the formation of TLS may bring new breakthroughs in EC treatment. For example, it is important to investigate how immunomodulators interact with the TME specific to EC and how they enhance TLS formation and function by modulating local immune responses in EC. Extensive experiments concerned with TLS in EC will be conducted in cell culture models, animal models and preclinical studies to assess the effectiveness and safety of these prospective mechanisms. It is reasonable to believe that these studies will provide new TLS-based strategies for the treatment of EC, especially in terms of improving immunotherapeutic efficacy and patient survival. In terms of innovations in clinical application strategies, in order to translate TLS research into practical applications for the treatment of EC, new diagnostic tools need to be developed to evaluate the existence and operational characteristics of TLS in EC. For example, the development of imaging-based techniques (e.g., PET-CT labeling techniques) for non-invasive monitoring of dynamic changes in TLS and its response to therapy. In addition, individualized treatment strategies based on TLS characteristics, for example, modifying the administration of immune checkpoint inhibitors based on the cellular composition of TLS in EC, may improve the targeting and efficacy of treatment. The future development of TLS research in EC will also depend on the integrated application of multidisciplinary techniques such as bioinformatics, systems biology and computational biology. These technologies can help integrate and analyze big data from genomes, transcriptomes, proteomes and clinical data to reveal the complex relationship between TLS and EC, as well as identify new biomarkers and therapeutic targets.

## 5 Discussion

In the context of EC treatment and investigation, TLS serves as a pivotal component of the immune landscape, facilitating a robust anti-tumor immune response. In EC, TLS emerges within the TME, serving as a critical site for the mobilization of immune cells, especially T and B cells. These cells contribute to the generation and maintenance of immune responses by forming structured lymphoid tissues. The occurrence of TLS within tumors correlates with patient outcomes across various cancer types, particularly EC. Studies have indicated that individuals exhibiting a high density and advanced maturation of TLS in EC experience extended OS and a longer duration of DFS (36, 83). This phenomenon may be due to the highly active immune environment in TLS that promotes more effective anti-tumor immune surveillance and attack. An example of this can be seen in the pronounced presence of high-density CD8+ T cells and B cells within TLS, which correlates closely with improved clinical outcomes. Further studies revealed that TLS is not only a naturally occurring product of immune response but its formation and function are also regulated by numerous cytokines and chemokines, such as CXCL13 and CCL21 (92–94). These factors play a decisive role in attracting immune cells to the TLS and promoting their organization within the region. In addition, the activation of DCs and other antigen-presenting cell types within the TLS is essential for activating and maintaining T cell and B cell responses (95). In EC, the characteristics of TLS are crucial not only for the assembly and activation of immune cells but also for its capacity to target and destroy tumor cells. And this is especially evident in the context of therapies that modulate immune checkpoints, such as those involving PD-1 and PD-L1 blockade. The existence of TLS in EC creates an optimal microenvironment that enhances the efficacy of therapeutic approaches. This phenomenon elucidates the increased likelihood of EC rich in TLS responding favorably to immunotherapy.

From a clinical perspective, the findings of TLS have a profound impact on the treatment of EC. Understanding the mechanism of TLS formation and its role in EC can not only help predict patients’ response to treatment and efficacy but may also directly influence treatment decisions. For example, the assessment of TLS in EC can be an important factor in determining a patient’s suitability for immunotherapy, particularly in the context of utilizing immune checkpoint inhibitors. Furthermore, considering the properties of TLS, we can design therapeutic regimens more precisely, for example, by facilitating the development and activity of TLS to strengthen the immune response in EC. Some novel therapeutic approaches, such as immunomodulators including pro-inflammatory factors and cytokine modulators, have shown potential in preclinical models to enhance therapeutic efficacy. These agents amplify the immune activity of TLS (96). Specifically, firstly, we may target PD-1, ICOS, or block the interaction between Tregs and CD20+IgD-CD27- B cells to enhance the anti-tumor effects of TLS. Secondly, combining CXCL13 agonists with PD-1 inhibitors to promote TLS maturation and reverse immune suppression could be a promising avenue for clinical translation. Thirdly, we could consider targeting antigen-presenting genes such as *HLA-A* to improve the antigen presentation efficiency within TLS of EC. Fourthly, we may consider combining PD-1 and CTLA4 blockade while activating the TNFRSF9 pathway to reverse T cell exhaustion within TLS of EC. Fifthly, developing targeted drugs against inhibitory cells such as PD-L1+ macrophages and Collagen I+ fibroblasts within TLS could also be a potential clinical strategy to improve immune therapy efficacy in EC patients. Sixthly, we believe that identifying immunomodulatory agents that promote the expansion and migration of CD39+ Tpex cells within TLS may become a new strategy for optimizing ICB efficacy. Seventhly, targeting the Collagen I-IL-10 axis may reverse the immunosuppressive phenotype of TLS and enhance immune therapy efficacy in EC. Such strategies, if successfully translated into clinical applications, may substantially improve outcomes in EC. The formation of TLS in EC is not only dependent on cell types and cytokines in the TME but is also influenced by tumor-specific gene expression patterns (44). New gene-targeted therapies could modulate these genes to create a TLS-favorable microenvironment in EC. This approach may ultimately enhance immunotherapy efficacy. The mechanism of action of TLS in EC is similar to that in other types of cancers; however, TLS in EC holds unique significance. To begin with, TME in EC contains a higher number of M2 macrophages and Tregs, leading to a more pronounced immunosuppressive environment (97). In EC, TLS acts as both an anti-tumor immune “sentinel” and a modulator of the immunosuppressive TME. It enhances anti-tumor effects and improves prognosis, distinguishing EC from cancers with higher TME anti-tumor cell infiltration. Additionally, in EC, TLS increases in response to the combination of PD-1 inhibitors compared to chemotherapy alone (86). Therefore, compared to cancers with lower sensitivity to PD-1 and PD-L1 inhibitors such as pancreatic cancer (98) and hepatocellular carcinoma (99), PD-1 inhibitor therapy for EC holds value in promoting TLS generation and maturation. Furthermore, in contrast to cancers like non-small cell lung cancer, where therapeutic strategies have advanced in addressing resistance to PD-1 and PD-L1 inhibitors, although ICB shows some efficacy in EC, its overall response rate remains relatively low and resistance is still a significant issue (100, 101). Therefore, TLS stands out as a promising new target for immunotherapy in EC, offering immense potential. In summary, our investigation into TLS within EC highlights its critical role in the tumor immune microenvironment and establishes a foundation for devising novel therapeutic approaches. Further mechanistic investigations and clinical trials are anticipated to establish TLS as a pivotal target in the treatment of EC, thereby offering patients more tailored and effective therapeutic options.

## Glossary

EC: Esophageal cancer
ESCC: Esophageal squamous cell carcinoma
TLS: Tertiary lymphoid structure
DCs: Dendritic cells
pCR: pathological complete response
mPFS: median progression-free survival
OS: Overall survival
TME: Tumor microenvironment
NK: Natural killer
TILs: Tumor-infiltrating lymphocytes
Tregs: Regulatory T cells
TLSs: Tertiary lymphoid structures
CD: Cluster of Differentiation
IFN-γ: Interferon-γ
TNF: Tumor necrosis factor
DFS: Disease-free survival
IL: Interleukin
CXCL C-X-C: motif chemokine ligand
Foxp3: Forkhead box protein 3
Tfh: Follicular helper T
CCL: Chemokine ligand
PD-L1: Programmed cell death 1 ligand 1
ICOS: Inducible synergistic co-stimulation molecules
PD-1: Programmed cell death protein 1
CXCR: CXC-chemokine receptor
Th: Helper T
GCs: Germinal centers
VCAM: Vascular cell adhesion *p*rotein
TLOs: Tumor-associated lymph nodes
mIHC: Multiple immunohistochemical staining
SLOs: Secondary lymphoid organs
HEVs: High endothelial microvessels
VCAM-1: vascular cell adhesion molecule-1
ICAM-1: intercellular cell adhesion molecule-1
LTi: Lymphoid tissue-inducing
LTα1β2: Lymphotoxin α1β2
LTβR: Lymphotoxin β receptors
PNAd: Peripheral node addressin
VEGF: Vascular endothelial growth factor
FDCs: Follicular dendritic cells
RFS: Recurrence-free survival
CCR: Chemoattractant cytokine ligand
MHC: Major histocompatibility complex
CTLA4: Cytotoxic T-lymphocyte-associated protein 4
HLA: Human leukocyte antigen
TNFRSF: Tumor necrosis factor receptor superfamily
TNFSF: tumor necrosis factor superfamily member
ICB: PD-1 blockade therapy
TCF: T Cell Factor
